# Mortality, intensive care unit admission, and ventilatory support in pregnant and nonpregnant women with COVID-19 in Brazil: a paired observational study

**DOI:** 10.1590/1980-549720250054

**Published:** 2025-11-21

**Authors:** Ana Elisa Madalena Rinaldi, Camila Abadia Rodrigues Meira, Marília Neves Santos, Luciana Bertoldi Nucci, Carla Cristina Enes, Wolney Lisboa Conde

**Affiliations:** I Universidade Federal de Uberlândia – Uberlândia (MG), Brazil.; II Pontifícia Universidade Católica de Campinas – Campinas (SP), Brazil.; III Universidade de São Paulo – São Paulo (SP), Brasil.

**Keywords:** Pregnant people, COVID-19, Severe acute respiratory syndrome-related coronavirus, Vaccination, Gestantes, COVID-19, Coronavírus relacionado à síndrome respiratória aguda grave, Vacinação

## Abstract

**Objective:**

To compare mortality, intensive care unit (ICU) admission, and use of invasive ventilatory support in hospitalized pregnant and nonpregnant women with severe acute respiratory syndrome due to Coronavirus-2 in Brazil.

**Methods:**

It is a cross-sectional study developed with data from the Brazilian Surveillance of Severe Acute Respiratory Syndrome (SARS), Ministry of Health. Three outcomes were investigated: mortality, ICU admission, and use of invasive ventilatory support among COVID-19 cases. Hospitalized pregnant and nonpregnant women were paired according to age group, geographic region, and epidemiological week (33,113 nonpregnant women; 15,567 pregnant women) from March 2020 to March 2022. Associations between pregnancy status and outcomes were analyzed using conditional logistic regression. For 2021 data, an interaction term with vaccination status (no/yes) was included.

**Results:**

Mortality (17.6 *vs* 7.9%), ICU admission (30.3 *vs* 26.1%), and invasive ventilatory support use (46.6 *vs* 38.5%) were higher among nonpregnant women, respectively. ICU admission was highest in the second trimester of pregnancy (32.6%). Being in the second trimester increased the likelihood of ICU admission (OR=1.26; 95%CI 1.15–1.39) compared to nonpregnant women. The odds of ICU admission was lower among vaccinated pregnant women in the first (OR=0.71; 95%CI 0.51–0.96), second (OR=0.74; 95%CI 0.62–0.88), and third trimesters (OR=0.65; 95%CI 0.57–0.74).

**Conclusion:**

All three outcomes were more frequent among hospitalized nonpregnant women, except for ICU admission in the second trimester. COVID-19 vaccination has proven to be an important protective measure, particularly for pregnant women in the second and third trimesters.

## RESUMO

**Objetivo:**

Comparar mortalidade, admissão em unidade de terapia intensiva (UTI) e uso de suporte ventilatório invasivo em mulheres gestantes e não-gestantes hospitalizadas com síndrome respiratória aguda grave causada pelo COVID-19 no Brasil.

**Métodos:**

Estudo transversal desenvolvido com dados do Sistema de informação da Síndrome Respiratória Aguda Grave (SRAG) do Ministério da Saúde. Três desfechos foram investigados: mortalidade, admissão em UTI e uso de suporte ventilatório invasivo entre casos de COVID-19. Mulheres hospitalizadas, gestantes e não-gestantes, foram pareadas por faixa etária, região geográfica e semana epidemiológica (33.113 não-gestantes; 15.567 gestantes) entre março de 2020 e março de 2022. As associações entre *status* gestacional e desfechos foram analisadas por regressão logística condicional. Para os dados de 2021, foi incluído um termo de interação com *status* vacinal (sim/não).

**Resultados:**

Mortalidade (17,6 *vs.* 7,9%), admissão em UTI (30,3 *vs.* 26,1%) e uso de suporte ventilatório invasivo (46,6 *vs.* 38,5%) foram mais elevados entre mulheres não-gestantes, respectivamente. A admissão em UTI foi maior no segundo trimestre da gestação (32,6%). Estar no segundo trimestre aumentou a chance de admissão em UTI em comparação às não-gestantes (OR=1,26; IC95% 1,15–1,39). A chance de admissão em UTI foi menor entre as gestantes vacinadas no primeiro (OR=0,71; IC95% 0,51–0,96), segundo (OR=0,74; IC95% 0,62–0,88), e terceiro trimestre (OR=0,65; IC95% 0,57–0,74).

**Conclusão:**

Os três desfechos foram mais frequentes entre mulheres não-gestantes hospitalizadas, com exceção da admissão em UTI no segundo trimestre. A vacinação contra a COVID-19 mostrou ser uma importante medida protetiva, especialmente para gestantes nos segundo e terceiro trimestres gestacionais.

## INTRODUCTION

At the beginning of the COVID-19 pandemic, the obstetric population was not considered an at-risk group^1^. However, as the pandemic advanced, studies worldwide^2–5^ began to identify that pregnant women had an increased risk of complications and admission to the intensive care unit (ICU), possibly due to physiological changes inherent to pregnancy, mainly cardio-respiratory and immune, which increase susceptibility to several infectious agents^6^.

Maternal deaths seemed more frequent in low- and middle-income countries during COVID-19 pandemic and were associated with serious failures in the health system, combined with the social determinants of the health-disease process. This has also been observed in other Latin American countries^7–12^, especially Mexico, which maintains an efficient notification system^13^.

Roberton et al.^14^ have already estimated, using the Lives Saved Tool, an increase in maternal mortality in 118 low- and middle-income countries, resulting from high birth rates, lack of investment, service disruption, and restructuring due to COVID-19. In Brazil, a concerning trend emerged, showing a rise in maternal deaths from COVID-19.

Takemoto et al.^8^ identified 124 maternal deaths, corresponding to a case fatality rate of 12.7% among COVID-19 acute respiratory distress syndrome cases in this obstetric population, which is higher than those reported thus far in the literature^2,15,16^. Rates may be even higher due to underreporting, difficulties in performing laboratory tests, possible false-negative results, and dependence on the health policies adopted by each region or country.

Brazil has the highest number of maternal deaths from COVID-19 in the Americas, with a lethality rate of 7.2%, more than double the country's current lethality rate of 2.8%^17^. Until January 2024, 2,075 maternal deaths had been registered, representing a fourfold increase from 2020, when 544 deaths of pregnant and postpartum women due to COVID-19 were reported in the country^17^.

Despite the growing number of studies on COVID-19 in pregnant women, there remains a gap in the literature regarding direct comparisons between hospitalized pregnant and nonpregnant women, particularly in low- and middle-income countries. A systematic review^18^ highlights that maternal mortality due to COVID-19 varies significantly across regions and income levels. However, it emphasizes the lack of high-quality individual-level data and the absence of studies evaluating other clinical outcomes beyond mortality, such as ICU admission and the use of ventilatory support, as well as the role of vaccination in reducing these outcomes.

A summary of the main analysis of COVID-19 in Pregnancy in Scotland (COPS) underscores the importance of studying early infection, vaccination rates among pregnant women, varying risk levels for maternal critical care based on COVID-19 variants, and the very low rate of infection among neonates during 2020-2021. The main outcomes of these reviews indicate the need to improve surveillance and readiness for pandemic events^19^.

Thus, this study aimed to compare mortality, ICU admission, and the use of invasive ventilatory support in hospitalized pregnant and nonpregnant women with severe acute respiratory syndrome due to Coronavirus-2 in Brazil.

## METHODS

### Data source and study population

The data were extracted from the Influenza Epidemiological Surveillance Information System (*Sistema de Informação da Vigilância Epidemiológica da Gripe* — SIVEP-Gripe), which serves as the official system for recording all cases and deaths of severe acute respiratory syndrome (SARS). Since the Influenza A (H1N1) pandemic, the Health Surveillance Department of the Brazilian Ministry of Health has carried out surveillance of SARS and, as of 2020, incorporated the surveillance of coronavirus infections^20^, following the Declaration of a Public Health Emergency of National Importance (*Emergência em Saúde Pública de Importância Nacional* — ESPIN) due to human infection by the coronavirus. This open data source represents all notified cases of SARS, covering both public and private health care settings nationwide. At the beginning of 2022, SARS-CoV-2 was added to the mandatory reporting list of diseases, conditions, and public health events for both public and private health care settings.

The first stage involved downloading the datasets for each year (2020, 2021, and 2022) in Excel format, converting them to Stata format, merging all datasets into a single file, analyzing the available variables, and identifying the presence of missing values. The distribution of missing values for selected variables was analyzed before matching and was similar for nonpregnant and pregnant women. The databases are organized in Excel and are available for download at: https://dados.gov.br/dados/conjuntos-dados/srag-2021-e-2022.

**Figure 1:**
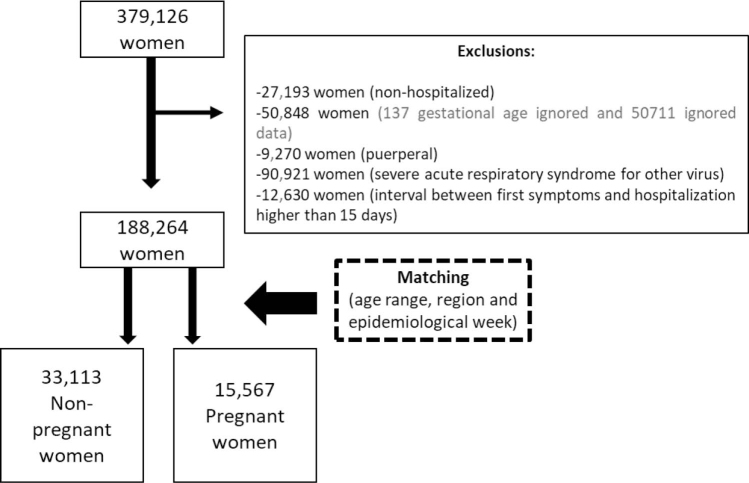
Flowchart of hospitalized nonpregnant and pregnant women selection from March, 2020 to March, 2022. Brazil, 2020-2022.

This study included notified SARS cases from March 2020 to March 2022. The eligibility criteria were women of childbearing age (15 to 49 years) and all hospitalized cases with a confirmed diagnosis of SARS due to COVID-19. Exclusion criteria were nonhospitalized women, women with missing data on pregnancy status (yes/no), women with an ignored answer for gestational status, puerperal women, SARS cases from other viruses, and cases with an interval greater than 15 days between symptom onset and hospitalization. The flowchart of the target population selection process is described in Figure 1. This study was not submitted for ethical assessment, as it used a public database with preserved anonimity.

#### Data availability statement:

Data available upon request to the corresponding author. The raw data are available in Excel databases on the website: https://dados.gov.br/dados/conjuntos-dados/srag-2021-e-2022. The results found can be obtained from the description of the methods used in our study.

### Outcome and exposure variables

The three outcomes analyzed were: death from COVID-19 (no/yes), ICU admission (no/yes), and invasive ventilatory support (IVS) in the ward and the ICU (no IVS, noninvasive respiratory support, and IVS). These outcomes refer to hospitalization data available in the information system. Ventilatory support and ICU admission are indicators of disease severity, with significant impact on maternal and child health. During the COVID-19 pandemic, the availability of ICU beds and ventilatory support was a critical factor in highly complex services and represented the resources required to meet the specific needs of patients with the disease.

The two main exposures were pregnancy status (pregnant or nonpregnant women) and gestational trimester (first, second, and third). Vaccine status (vaccinated/unvaccinated) was analyzed for data from 2021 and 2022. In this study, women were classified as vaccinated if they had received at least one dose of any vaccine, regardless of type.

### Data analysis

The number of nonpregnant women in the dataset was more than ten times higher than that of pregnant women. Pregnant women were younger than nonpregnant women, and their percentage was higher in the North, Northeast, and Central-West (Table 1). Therefore, all analyses were performed on a matched dataset of pregnant and nonpregnant women. Matching was conducted using three variables: age, geographic region, and epidemiological week of notification. Nonpregnant women were randomly selected to achieve a ratio of two nonpregnant women for each pregnant woman across all matching strata.

**Table 1:** Demographic data, death, type of respiratory support use received in hospitals and vaccination for hospitalized nonpregnant and pregnant women before matching. Brazil, 2020–2022.

		Nonpregnant women (n=172,697) % (95%CI)	Pregnant women (n=15,567) % (95%CI)
Age range (years)			
	15 to 19	1.7 (1.6–1.8)	6.8 (6.4–7.2)
	20 to 24	4.3 (4.2–4.4)	17.1 (16.4–17.7)
	25 to 29	8.2 (8.0–8.3)	23.2 (22.4–23.9)
	30 to 34	13.0 (12.9–13.2)	25.0 (24.3–25.7)
	35 to 39	19.5 (19.3–16.7)	19.7 (19.0–20.4)
	40 to 44	24.9 (24.7–25.1)	6.6 (6.2–7.0)
	45 to 49	28.4 (28.2–28.6)	1.6 (1.3–1.8)
Region			
	North	7.6 (7.5–7.8)	10.8 (10.3–11.4)
	Northeast	14.6 (14.5–14.8)	20.8 (20.1–21.5)
	Central-West	10.3 (10.2–10.5)	13.4 (12.8–14.0)
	Southeast	48.0 (47.8–48.3)	39.6 (38.7–40.4)
	South	19.3 (19.2–19.6)	15.3 (14.7–16.0)
Death			
	No	82.4 (82.2–82.6)	90.9 (90.3–91.4)
	Yes	17.6 (17.4–17.8)	9.1 (8.6–9.7)
ICU			
	No	70.4 (70.2–70.6)	71.8 (71.0–72.6)
	Yes	29.6 (29.4–29.8)	28.2 (27.4–29.0)
Respiratory support use			
	No	25.2 (25.0–25.5)	47.5 (46.6–48.5)
	Yes	74.8 (74.5–75.0)	52.5 (51.5–53.4)
Vaccination (2021)*			
	No	91.9 (91.7–92.0)	93.7 (93.2–94.2)
	1 dose	6.4 (6.2–6.5)	4.6 (4.1–5.0)
	2 doses	1.8 (1.7–1.9)	1.7 (1.5–2.0)
		**Mean (95%CI)**	**Mean (95%CI)**
Time interval between first symptoms and hospitalization (days)		7.0 (6.9–7.0)	First trimester: 5.7 (5.5–5.9) Second trimester: 6.3 (6.1–6.4) Third trimester: 5.4 (5.3–5.5) Unknown: 5.3 (5.0–5.7)
Stay in ICU (days)		1.2 (1.1–1.3)	First trimester: 1.0 (0.6–1.4) Second trimester: 1.0 (0.6–1.4) Third trimester: 1.2 (1.1–1.3) Unknown: 0.8 (0.6–1.1)

* n=118,452 ICU: intensive care unit

The three outcomes (death, ICU admission, and IVS) were described according to pregnancy status (pregnant or nonpregnant women) and gestational trimester as percentages with 95% confidence intervals (95%CI). The interval (days) between symptom onset and hospitalization and between hospitalization and ICU admission were estimated as means with 95%CI. Comparisons of percentages between nonpregnant and pregnant women were assessed by overlap of the 95%CI. Conditional logistic regression was used to assess the association between pregnancy status (pregnant and nonpregnant women), gestational trimester, and the three outcomes. The association measure used was the odds ratio (OR) with the respective 95%CI. Conditional logistic regression differs from regular logistic regression in that the data are matched and the likelihood is calculated relative to each group. For data from 2021 and 2022, an interaction term between pregnancy status and vaccination status was included in the conditional logistic regression. The OR of outcomes by vaccination status was tested for pregnant and nonpregnant women, and the OR values were lower among vaccinated pregnant women. Therefore, an interaction term was included to analyze the modified likelihood of outcome frequency due to vaccination. The significance level adopted was 5%. All analyses were performed using Stata SE 15.0.

## RESULTS

### Sample description

The demographic data and outcomes of nonpregnant and pregnant women before matching are described in Table 1. Pregnant women were younger than non-pregnant women, and their proportion was higher in the North, Northeast, and Central-West. Death frequency and respiratory support use were higher among nonpregnant women. Vaccination percentages were low in both groups. The mean interval between symptom onset and hospitalization was 7.0 days for nonpregnant women and ranged from 5.3 to 6.3 days for pregnant women, depending on the gestational trimester. The mean time of ICU admission was approximately 1 day for both pregnant and nonpregnant women (Table 1).

### Prevalence of outcomes

Table 2 presents the prevalence of death, ICU admission, and respiratory support use (invasive or noninvasive) among non-pregnant and pregnant women. The prevalence of death was more than twice as high in nonpregnant women (17.6%) compared with pregnant women (7.9%). Among pregnant women, death prevalence was highest in the second trimester (10.0%). ICU admission was more frequent in non-pregnant women (30.3%) and, among pregnant women, in the second trimester (35.6%). Regarding ventilatory support use in the ward, the percentages of both noninvasive and invasive support were higher in nonpregnant women. However, in the second trimester of pregnancy, the percentage of noninvasive respiratory support use was the highest compared with other gestational trimesters and with nonpregnant women. In the ICU, the percentage of IVS was higher in non-pregnant women (46.6%) compared with pregnant women (38.5%).

**Table 2:** Prevalence of death, intensive care unit admission, and type of respiratory support use in the ward and intensive care unit by hospitalized nonpregnant and pregnant women and the gestational trimester after matching. Brazil, 2020–2022.

		Death	Intensive care unit admission	Ward			Intensive care unit admission		
				Without respiratory support	Noninvasive respiratory support	Invasive respiratory support	Without respiratory support	Noninvasive respiratory support	Invasive respiratory support
		Prevalence (95%CI)								
Nonpregnant women (n=172,697)		17.6 (17.2–18.1)	30.3 (29.7–30.8)	34.5 (33.8–35.2)	62.5 (61.8–63.2)	3.0 (2.8–3.3)	9.4 (8.7–10.0)	44.1 (43.0–47.3)	46.6 (45.5–47.7)
Pregnant women (all trimesters) (n=15,567)		7.9 (7.5–8.4)	26.1 (25.3–26.8)	64.1 (63.1–65.1)	34.4 (33.5–35.4)	1.5 (1.2–1.7)	15.8 (14.6–17.2)	45.6 (44.0–47.3)	38.5 (36.9–40.2)
Gestational trimesters									
	1^st^ trimester	7.2 (5.9–8.8)	22.0 (19.8–24.5)	59.3 (56.0–62.7)	36.2 (33.1–39.4)	1.5 (0.01–2.5)	16.9 (12.8–22.1)	50.4 (44.2–56.6)	32.7 (27.1–38.7)
	2^nd^ trimester	10.0 (9.0–11.0)	32.6 (31.1–34.2)	51.5 (49.4–53.6)	44.4 (42.3–46.4)	1.9 (1.4–2.6)	14.3 (12.4–16.5)	47.3 (44.3–50.2)	38.4 (35.6–41.3)
	3^rd^ trimester	7.1 (6.5–7.6)	24.2 (23.3–25.1)	66.0 (64.7–67.3)	30.0 (28.8–31.1)	1.2 (1.0–1.6)	16.5 (15.0–18.3)	44.4 (42.2–46.6)	39.1 (36.9–41.3)
	Unknow gestational age	9.8 (7.6–12.5)	21.1 (17.8–24.9)	53.3 (47.9–58.7)	43.1 (38.0–48.4)	2.3 (1.1–4.5)	16.7 (10.1–26.2)	39.3 (29.5–50.1)	44.0 (33.8–54.8)

### Association with outcomes

First, all pregnant women were compared with nonpregnant women, and the odds of death were lower for pregnant women (OR=0.49; 95%CI 0.44–0.54) across all gestational trimesters. Next, pregnant women by gestational age were compared with nonpregnant women, and the odds of ICU admission were higher in the second trimester of pregnancy (OR=1.26; 95%CI 1.15–1.39). IVS in the ward was lower in the third trimester (OR=0.41; 95%CI 0.30–0.57), and IVS in the ICU was lower in the first trimester (OR=0.65; 95%CI 0.45–0.94), compared with nonpregnant women (Table 3).

**Table 3:** Association of pregnant status, gestational trimester and outcomes (death, intensive care unit, and invasive respiratory support) for hospitalized pregnant and nonpregnant women. Brazil, 2020–2022.

	Death	ICU Admission	Ward	ICU
			Invasive respiratory support	Invasive respiratory support
	OR (95%CI)			
Nonpregnant women (n=172,697)	1.00 (Ref)	1.00 (Ref)	1.00 (Ref)	1.00 (Ref)
Pregnant women (n=15,567)	0.49 (0.44–0.54)	0.98 (0.92–1.05)	0.52 (0.40–0.67)	0.85 (0.75–0.97)
1^st^ trimester	0.48 (0.37–0.63)	0.74 (0.62–0.89)	0.69 (0.37–1.29)	0.65 (0.45–0.94)
2^nd^ trimester	0.61 (0.52–0.69)	1.26 (1.15–1.39)	0.69 (0.46–1.03)	0.85 (0.71–1.02)
3^rd^ trimester	0.43 (0.38–0.48)	0.93 (0.87–1.01)	0.41 (0.30–0.57)	0.87 (0.75–1.01)
Unknow gestational age	0.64 (0.45–0.90)	0.70 (0.53–0.93)	0.89 (0.37–2.17)	1.25 (0.64–2.46)

ICU: intensive care unit.

### Interaction with vaccination

Pregnant women had a lower likelihood of death with or without immunization, and OR values were lower for vaccinated women in all gestational trimesters. Regarding ICU admission, immunization protected pregnant women in all trimesters. Among unvaccinated pregnant women, the odds of ICU admission were higher, particularly in the second trimester. The odds of IVS use in the ward were lower for unvaccinated pregnant women in the third trimester and for vaccinated nonpregnant women. The use of IVS in the ICU was similar for unvaccinated women and for vaccinated pregnant and nonpregnant women, compared with unvaccinated nonpregnant women (Table 4).

**Table 4:** Comparison of outcomes prevalence for hospitalized pregnant and nonpregnant women, by vaccines status interaction. Brazil, 2020–2022.

		Death	ICU Admission	Ward	ICU
				Invasive respiratory support	Invasive respiratory support
		OR (95%CI)			
Nonpregnant women/unvaccinated		1.00 (Ref)	1.00 (Ref)	1.00 (Ref)	1.00 (Ref)
Pregnant unvaccinated					
	1^st^ trimester	0.53 (0.41–0.70)	0.82 (0.68–0.98)	0.65 (0.32–1.31)	0.72 (0.49–1.06)
	2^nd^ trimester	0.66 (0.57–0.77)	1.38 (1.24–1.52)	0.73 (0.48–1.11)	0.88 (0.73–1.06)
	3^rd^ trimester	0.47 (0.42–0.53)	1.13 (1.04–1.23)	0.42 (0.30–0.60)	0.89 (0.77–1.04)
Nonpregnant vaccine		0.80 (0.74–0.86)	0.94 (0.87–1.01)	0.74 (0.59–0.93)	0.95 (0.85–1.04)
Vaccine*pregnant					
	1^st^ trimester	0.48 (0.23–1.01)	0.71 (0.51–0.96)	1.39 (0.58–3.36)	0.46 (0.17–1.26)
	2^nd^ trimester	0.37 (0.22–0.63)	0.74 (0.62–0.88)	0.77 (0.29–2.03)	0.70 (0.46–1.07)
	3^rd^ trimester	0.45 (0.32–0.63)	0.65 (0.57–0.74)	0.91 (0.50–1.68)	0.76 (0.55–1.03)

## DISCUSSION

The main results of this study highlight a higher percentage of hospitalized nonpregnant women experiencing death, ICU admission, and IVS use in both the ward and the ICU compared with pregnant women. Pregnant women were less likely to die if immunized. The second trimester of pregnancy was associated with the highest risk for ICU admission. This risk was mitigated in all gestational trimesters among pregnant women who had received at least one dose of the COVID-19 vaccine. Immunized nonpregnant women were less likely to require ventilatory support in the ward; however, such protection was not observed for pregnant women. Interestingly, the mean interval between the first symptoms of COVID-19 and hospitalization was shorter among pregnant women in the first and third trimesters. No differences were observed in ICU length of stay between pregnant and nonpregnant women.

Although pregnant women infected with COVID-19 are considered more vulnerable to viral infection^21–23^, severe disease is theoretically more likely to occur in the third trimester, as pregnancy alters the immune system and its response to viral infection^24^. However, the literature shows that disease progression, signs and symptoms, and severity of COVID-19 are similar in pregnant and nonpregnant women^25–27^. Thus, evidence regarding outcomes for pregnant women infected with COVID-19 remains inconsistent. This study is among the first to compare the impact of COVID-19 on the health of pregnant and nonpregnant women in Brazil. Previous studies have investigated unfavorable health outcomes among pregnant women infected with COVID-19 in both severe and non-severe forms of the disease^28,29^.

According to our analyses, the odds of death were lower in all gestational trimesters, consistent with a previous meta-analysis^30^. Literature findings are conflicting: some studies suggest pregnancy increases the risk of death^31–33^, while another study^34^ suggests that pregnancy is protective against death. When these studies were pooled in a meta-analysis^30^, pregnant women with COVID-19 did not show an increased risk of death compared with nonpregnant women. The quality of results in this meta-analysis is noteworthy, as sensitivity and subgroup analyses were performed on studies yielding robust results^30^. A possible explanation is that pregnant women receive higher-quality care for viral infections and may adopt more protective health behaviors than nonpregnant women of similar social and demographic backgrounds.

Regarding ICU admission, pregnant women in the second trimester had a higher risk compared with nonpregnant women, consistent with a meta-analysis showing pregnancy was associated with increased risk for ICU admission. However, that meta-analysis did not differentiate between gestational trimesters^30^. Unlike that study^30^, our results did not show an increased risk for ventilatory support. Two studies reported a lower risk of ICU admission compared with nonpregnant women^35,36^. Therefore, data on the clinical impact of COVID-19 in pregnant women remain inconsistent in the literature.

A higher risk of ICU admission was observed for pregnant women, likely due to the physiological and immunological changes that occur during pregnancy, which increase the risk or severity of certain infections^37^. During pregnancy, immunosuppression is maintained by inhibition of T-cell activity, making women more vulnerable to viral infection^38,39^. When pregnant women are infected with COVID-19, these immunological changes can impair viral clearance and worsen clinical outcomes, particularly in the third trimester^1,40–42^. The respiratory system is influenced by high estrogen and progesterone levels, changes in steroid hormones, and restricted lung expansion^43^. These conditions increase vulnerability to respiratory infections, which may progress to severe disease. Pregnancy is characterized by a pro-inflammatory state, especially in early and late stages, during which virus-induced cytokines can exacerbate inflammation^43^, posing risks to maternal and fetal health. Collectively, these alterations predispose pregnant women to more severe and prolonged forms of COVID-19^30^.

It is important to consider that ICU admission and the use of ventilatory support during the COVID-19 pandemic did not solely reflect clinical severity but were also influenced by resource availability and health service overload. At different stages of the pandemic, shortages of ICU beds, mechanical ventilators, and specialized staff may have limited access to intensive care for both pregnant and nonpregnant women. Studies conducted in the Brazilian context indicate that, during health system collapse, clinical decisions were influenced by prioritization criteria and immediate resource availability, which may have contributed to variations in the provision and use of advanced life support. Therefore, the outcomes analyzed in this study should also be interpreted in light of regional inequalities and the health system's response capacity, which affected equity of care during the pandemic^44^.

A protective effect of the vaccine for pregnant women was observed, even with a single dose. A study including pregnant women infected and non-infected with coronavirus from 18 countries, including Brazil, also demonstrated a protective effect of vaccination. The vaccine reduced severe symptoms, maternal death, and ICU admission, particularly among pregnant women who completed the vaccination schedule and received a booster dose. Protection was greater for overweight and obese pregnant women^45^.

Several strengths of this study should be noted. One was the use of a large and comprehensive database (SARS) from the Brazilian Ministry of Health, which includes all reported hospitalizations for SARS with COVID-19 confirmed by a highly sensitive and specific test (RT-PCR). This national database, available since 2008, provides robust information on SARS caused by different biological agents and allows monitoring of exposures during pregnancy.

Another strength was the comparison of the three outcomes between pregnant women and nonpregnant women. Most studies in the literature focus on comparisons between pregnant women infected and not infected with COVID-19, without including nonpregnant women. Additional strengths include the matching of the sample by factors that could influence outcomes, such as age, geographic region, and epidemiological week. Matching contributed to creating a more "homogeneous" sample and allowed assessment of the "effect" of vaccination between the two groups. Finally, the evaluation of vaccination status among both pregnant and nonpregnant women was also an important contribution.

This study has limitations. It did not adjust for missing data related pre-existing conditions other than COVID-19, such as comorbidities. It was not possible to determine whether ICU admission and IVS use among pregnant women occurred due to pregnancy itself or because of other serious health conditions associated with COVID-19 infection that required more intensive interventions. Additionally, 13.4% of women were excluded from the analysis due to missing information on pregnancy status, which may have introduced selection bias. These limitations are inherent to health service data, which are not designed as structured research instruments. Furthermore, the information was recorded during a pandemic, under high service demand and adverse conditions for healthcare professionals responsible for completing the information system.

We also did not adjust for confounders, such as sociodemographic characteristics (skin color and schooling) and comorbidities (obesity, diabetes, hypertension, and other diseases), due to the high percentage of missing data for both nonpregnant and pregnant women. Sociodemographic characteristics can influence both access to health services and the severity of outcomes, introducing potential confounding bias. Non-white women or those with less education may face additional barriers to timely care and vaccination, which could negatively affect the outcomes assessed. Potential selection bias should be considered, as about 7% of all non-hospitalized women were excluded from the original database, removing less severe cases treated in outpatient clinics. This exclusion may have led to an overestimation of outcome severity in the overall infected population. Therefore, extrapolation of our results should be limited to hospitalized women.

Another point to highlighting is the definition of vaccination based on at least one dose. This definition may not adequately reflect the degree of immunological protection, especially considering the different vaccination schedules available in Brazil during the analyzed period (single-dose *versus* multiple-dose vaccines). Thus, part of the observed protective effect may reflect differences in vaccine type, dosing intervals, and time since vaccination, factors not controlled for in this study but relevant for future analyses. Furthermore, the percentage of women who received multiple doses was very low (<2%).

These findings have important implications for public health practice in Brazil. Identifying a higher risk of ICU admission among pregnant women in the second trimester and demonstrating the protective effect of vaccination, even with a single dose, reinforces the importance of prioritizing immunization for this group, particularly in contexts of high transmissibility. Incorporating these findings into health policies may contribute to more effective maternal and neonatal protection strategies, including targeted vaccination campaigns, intensive monitoring of pregnant women in the second trimester, and strengthening obstetric surveillance in hospital units.

This study identified a higher risk of adverse outcomes in nonpregnant women, except in the second trimester of pregnancy. Vaccination reduced the likelihood of these outcomes, particularly among pregnant women. Further studies adjusting for confounding factors and examining additional health conditions, whether or not associated with COVID-19, are necessary to further validate these findings. Moreover, research assessing the long-term effects of COVID-19 among pregnant women is essential to better understand the extent of the protective effects identified in this study.
